# Comprehensive experimental datasets of quasicrystals and their approximants

**DOI:** 10.1038/s41597-024-04043-z

**Published:** 2024-11-13

**Authors:** Erina Fujita, Chang Liu, Asuka Ishikawa, Tomoya Mato, Koichi Kitahara, Ryuji Tamura, Kaoru Kimura, Ryo Yoshida, Yukari Katsura

**Affiliations:** 1 The Institute of Statistical Mathematics (ISM), Research Organization of Information and Systems, Tachikawa, Tokyo 190-8562 Japan; 2 National Institute for Materials Science (NIMS), Tsukuba, Ibaraki 305-0047 Japan; 3 Department of Materials Science and Technology, Tokyo University of Science, Katsushika- ku, Tokyo 125–8585 Japan; 4 Department of Materials Science and Engineering, National Defense Academy, Yokosuka, Kanagawa 239-8686 Japan; 5 Graduate University for Advanced Studies, SOKENDAI, Tachikawa, Tokyo 190-8562 Japan; 6 Graduate School of Science and Technology, Tsukuba University, Tennodai, Tsukuba, Ibaraki 305-8573 Japan; 7 RIKEN Center for Advanced Intelligence Project, RIKEN, Chuo-ku, Tokyo 103-0027 Japan

**Keywords:** Databases, Metals and alloys, Electronic properties and materials

## Abstract

Quasicrystals are solid-state materials that typically exhibit unique symmetries, such as icosahedral or decagonal diffraction symmetry. They were first discovered in 1984. Over the past four decades of quasicrystal research, around 100 stable quasicrystals have been discovered. In recent years, machine learning has been employed to explore quasicrystals with unique properties inherent to quasiperiodic systems. However, the lack of open data on quasicrystal composition, structure, and physical properties has hindered the widespread use of machine learning in quasicrystal research. This study involves a comprehensive literature review and manual data extraction to develop open datasets consisting of composition, structure types, phase diagrams, and sample fabrication processes for a wide range of stable and metastable quasicrystals and approximant crystals, as well as the temperature-dependent thermal, electrical, and magnetic properties.

## Background & Summary

Quasicrystals (QCs) are a class of aperiodic materials that typically possess unique symmetries, such as icosahedral or decagonal diffraction symmetry, which are different from those of ordinary crystals, and exhibit highly ordered atomic arrangements. QC was discovered in the Al-Mn alloy system by Dan Shechtman^1^ and named by Paul J. Steinhardt^2^ in 1984, which was a metastable quasicrystal obtained by rapid cooling of a liquid alloy. The first thermodynamically stable QC was found in the Al-Li-Cu system in 1986^3^. Following these discoveries, An-Pang Tsai discovered a stable and higher quality QC in the Al-Fe-Cu alloy system in 1987^4^. Owing to the continuous developments with regard to unraveling new QCs, in 1992, the International Union of Crystallography revised the definition of crystals to include QCs as a new form of crystalline materials^5^.

Since the first QC was unraveled by Dan Shechtman 40 years ago, more than 100 stable QCs have been found. Their quasiperiodic structures are classified into two-dimensional and three-dimensional categories^6^. The material discovered by Shechtman was an icosahedral three-dimensional quasicrystal (IQC). Three-dimensional quasicrystals are known to form an icosahedral structure. Two-dimensional quasicrystals are composed of dodecagonal QCs (DoQCs), decagonal QCs (DQCs), and octagonal QCs (OQCs). Crystalline systems where the same structural units as a particular QC type are periodically arranged are called approximant crystals (ACs). These are referred to by the structural type of the related quasicrystal, such as decagonal QC approximants (DACs) and icosahedral QC approximants (IACs).

The quasiperiodic materials exhibit distinct characteristics different from conventional periodic systems. For instance, conventional metals possess high electrical conductivity, with electrical resistivity tending to increase with temperature. Contrastingly, many QCs have low electrical conductivity, and their electrical resistivity decreases with increasing temperature^7,8^. Similarly, the temperature dependence of thermal conductivity in QCs and conventional metals shows opposite trends above room temperature^9^. The high-temperature specific heat of QCs is significantly higher than the Dulong–Petit value, which is the saturation value for conventional solids^10^. The physical mechanisms responsible for these temperature-dependent properties have been elucidated^11–13^. Recently, quantum critical^14^, superconducting^15,16^, and ferromagnetic^17,18^ QCs have been discovered. The variety of QCs unravels the essential differences in electronic states between QCs and conventional crystals. Further enhancements in QC variety, such as semiconducting, antiferromagnetic, ionic bonding, oxide QCs, and others, are expected to accelerate the understanding of QC characteristics.

Recently, the application of machine learning to the realm of quasicrystals has shown notable progress. Liu *et al*.^19^ developed a machine learning classifier to predict whether a stable phase resulting from any given composition constitutes a QC or an AC. The model was trained using the chemical compositions of the available QCs and ACs. Subsequently, in Liu *et al*.^20^, this classifier was leveraged for high-throughput virtual screening across extensive composition spaces, leading to the discovery of three QCs. Uryu *et al*.^21^ developed a binary classifier to determine the presence of IQC in a multi-phase sample based on its powder X-ray diffraction pattern, leading to a novel QC in the Al-Ru-Si system from data accumulated in their laboratory. These pioneering studies have demonstrated the potential of machine learning as a new tool for the exploration of novel QCs. Nonetheless, compared to other material systems, the application of machine learning in QC research is lacking. This is due to the absence of data resources. To date, there is no comprehensive repository of structural and property data for QCs and ACs comparable to those available for ordinary periodic crystalline materials such as ICSD^22^, Materials Project^23^, AFLOW^24^, OQMD^25,26^ and AtomWorks^27^.

In this study, we systematically constructed an open dataset of QCs and ACs, called HYPOD-X (Hypermaterials Open Datasets for X, where X represents a wildcard for application targets, such as machine learning), through a comprehensive literature survey and data extraction. The atomic configurations of QC and AC can be described in a unified manner by projection from a higher-dimensional periodic lattice into three-dimensional space^6^. In our scientific project (https://www.rs.tus.ac.jp/hypermaterials/en/index.html), we refer to QCs and ACs as hypermaterials, a class of materials that can be regarded as “high-dimensional periodic crystals”. This category also includes incommensurately modulated structures and incommensurate composites. The composition dataset encompasses 915 QCs, 525 ACs, and 8 QCs or ACs synthesized to date, along with their structural types, sample preparation methods, and the corresponding bibliographic information. Additionally, we curated a dataset of phase diagrams by digitizing regional information from 43 ternary alloy phase diagrams extracted from images in journal articles. Furthermore, we compiled a dataset of temperature-dependent physical properties, including electrical resistivity, Seebeck coefficient, thermal conductivity, and magnetic susceptibility for 925 quasiperiodic materials. All these data are structured and distributed in machine-readable text formats.

## Methods

**Fig. 1:**
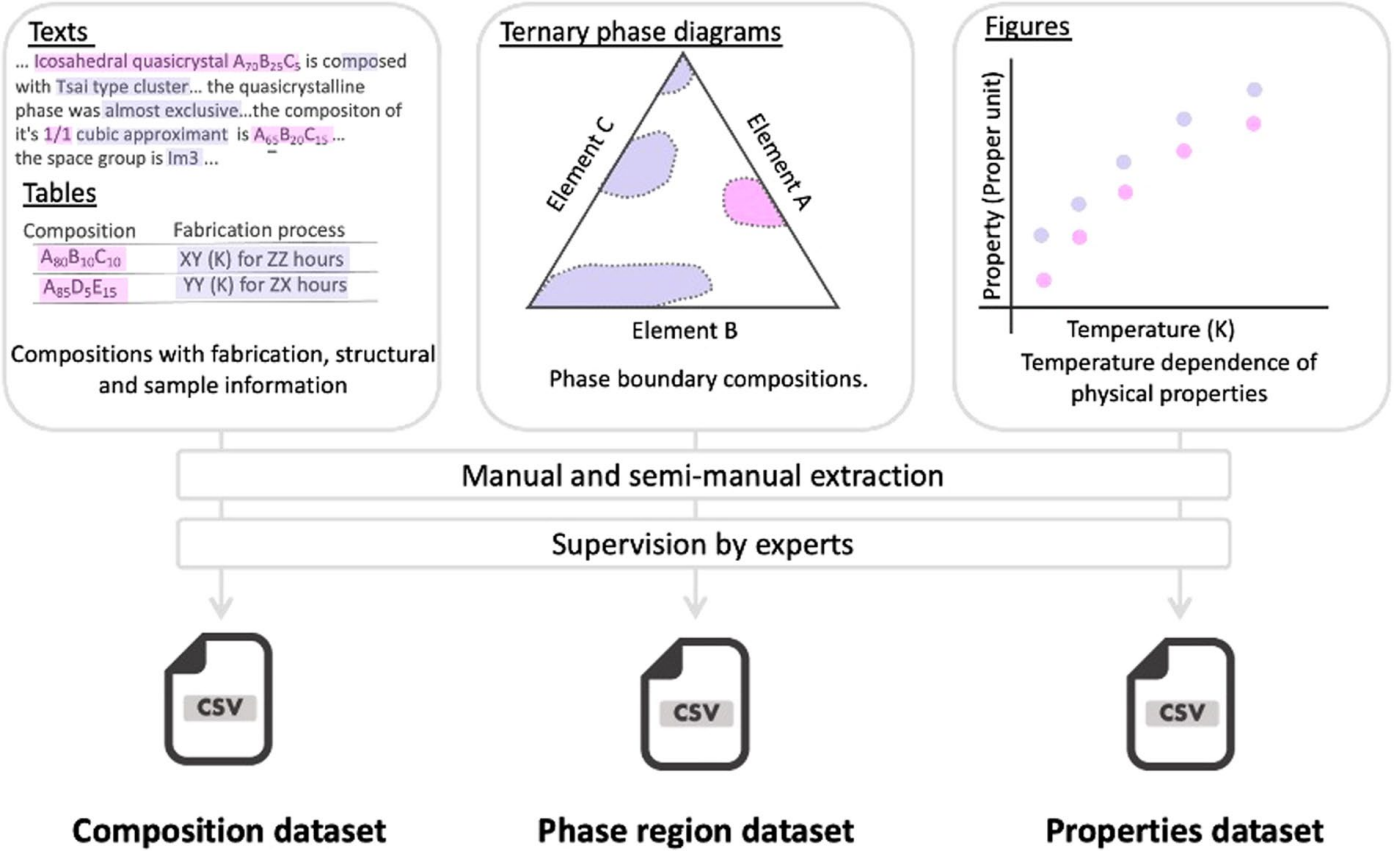
Workflow for extracting data from journal articles.

Data were manually extracted from literature in a variety of formats, including text, tables, and figures. In-house software was used to facilitate the data collection process along with our web application, Starrydata2 (https://www.starrydata2.org/)^28^. Additionally, an open web application called WebPlotDigitizer^29^ was employed to digitize images (Fig. 1). These digitized data, consisting of composition, phase diagram, and properties datasets, are distributed on Figshare^30^.

### Composition dataset

The composition dataset was constructed by compiling data described in text or tables in 130 journal articles. The validity of the textual information was carefully and rigorously reviewed by three experts. Each compositional piece of information was recorded on a single line. The data were saved and provided in the form of a comma-separated values (CSV) file.

QCs and ACs are classified as IQC, IAC, DQC, or DAC based on their structural categories. ACs are further classified according to the degree of approximation to the corresponding QC, such as 1/0, 1/1, 2/1, and 3/2^6^. As another classification criterion, IQCs and their IACs are classified into the Mackay, Bergmann, or Tsai cluster according to their basic structural unit, consisting of a few dozen atoms, arranged to approximate an icosahedron cluster^6^. In an IQC, clusters are geometrically arranged into a simple cubic (P-type), body-centered cubic (I-type), or face-centered cubic (F-type) type quasi-lattice in six-dimensional space. Other structural information such as space groups and lattice parameters are also provided where indicated in the literature.

The dataset provides labels indicating the stability of each material, denoted as “stable” or “metastable” in terms of thermodynamics. Detailed information on the heat treatment during the sample fabrication process was also described in “heat treatment” and “heat treatment condition” columns. In addition, each sample was labeled to distinguish whether it was observed in a single- or multi-phase in the “phase information” column. Samples with the same composition but different fabrication procedures were distinctly considered and recorded separately. Additionally, the compositions recorded in the “composition types” were categorized as “nominal”, “alloy”, and “analyzed”, respectively. For the “analyzed” compositions, details on analytic methods were also described, if available. If the composition was given as an interval in the original journal articles (e.g. A_80_B_20-y_C_y_, 0 < y < 20), the data were duplicated with equally spaced grid points within the interval. For the reference information, the composition that was described as a main result in the literature is referred to as “main”, and that only cited is referred to as “reference.”

In summary, the dataset contains a wide variety of attribute information associated with the compositional information. The complete list of attributes is described in the Data Records section.

### Phase region dataset

Currently, the phase region dataset records the phase diagrams of 43 aluminum (Al) ternary systems. Since the discovery of quasicrystal in the Al-Mn alloy system, Al alloys have been one of the most actively studied systems in QC research. Around 1990, Tsai *et al*. uncovered a series of stable QCs in Al-Cu-Fe and Al-Pd-Mn alloys. Subsequently, since the early 2000s, Benjamin Grushko determined the ternary phase diagrams of numerous Al-based quasicystalline and approximant crystal alloys. We extracted regional information regarding the quasicrystalline, approximant, liquid, and ordinary crystalline phases from the phase diagrams by Grushko. WebPlotDigitizer^26^ and an Excel macro written in Visual Basic for Applications (VBA) were used to convert the coordinates of the boundary for each phase region extracted from the phase diagram. In-house tools created using Python were used to perform the post-processing of the extracted data (e.g., merging individually separated columns into one dict type variable).

First, using WebPlotDigitizer (version 4.4), three vertices at the corners of a ternary phase diagram were captured, and their image coordinates, along with composition values, were defined as the reference point set. In addition, two orthogonal axes were specified in the image space for automated extraction of the coordinate value of any clicked point. Subsequently, the outer boundary of each phase region was traced by successively clicking on it. Consequently, the outer point set of each phase region was recorded as a set of x-y coordinates. These data were temporarily stored in WebPlotDigitizer and subsequently downloaded as a CSV file after tracing all phases in the diagram.

Note that the extracted boundary points form a coordinate set in Euclidean space; therefore, it is necessary to further transform them into their composition values. To address this, we used a VBA macro that implements a custom-made coordinate transformation algorithm. Let ${u}_{0},\,{u}_{1},\,{u}_{2}$ be the two-dimensional coordinate vectors in the reference set on the corners of the phase diagram image that were extracted as described above. Let ${c}_{0},\,{c}_{1}$, and $\,{c}_{2}$ be their known composition values. The transformation from $u$ to $c$ is linearly described as follows:

$$c={Mu}+v$$

Here, $M$ and $v$ must be determined to define the mapping from $u$ to $c$. The difference between any two compositions, e.g. ${c}_{1}-{c}_{0}$, is related to their extracted coordinates independent of the origin, as follows:

$$\left({c}_{1}-{c}_{0}\right)=M\left({u}_{1}-{u}_{0}\right)$$

Expressing the two equations for $\left({c}_{1},\,{u}_{1}\right)$ and $\left({c}_{2},\,{u}_{2}\right)$ in matrix form yields the following expression:

$$\left[{c}_{2}-{c}_{0},\,{c}_{1}-{c}_{0}\right]=M\left[{u}_{2}-{u}_{0},\,{u}_{1}-{u}_{0}\right]$$

The solution for the transformation matrix $M$ is then given as:

$$M=\left[{c}_{2}-{c}_{0},\,{c}_{1}-{c}_{0}\right]{\left[{u}_{2}-{u}_{0},{u}_{1}-{u}_{0}\right]}^{-1}$$

Finally, the intercept term can be estimated as:

$$v={c}_{0}-M{u}_{0}$$

**Fig. 2:**
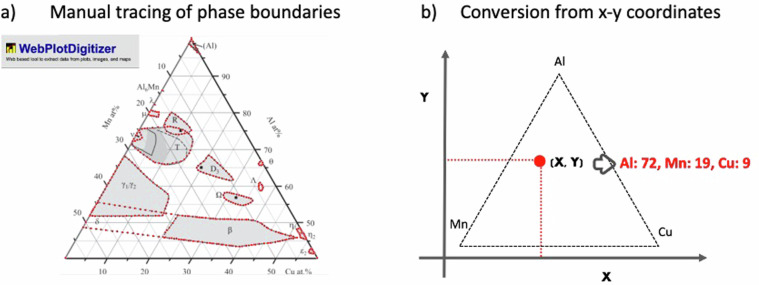
(**a**) Original image of an Al-Cu-Mn alloy system on the WebPlotDizitizer screen. B. Grushko, *et al*.^28^ was copied and pasted. (This figure is presented with permission for reuse by Elsevier.) Subsequently, phase boundaries were manually traced by clicking. Points are shown as red dots (**b**). Extracted coordinates were converted into element ratios via a VBA macro.

In our workflow, the extracted image coordinates in the phase diagram (Fig. 2a) were downloaded as a CSV file, and then transformed into the composition values by using the VBA macro (Fig. 2b).

### Properties dataset

The properties dataset was constructed by extracting temperature-varying physical property values of QCs and ACs from 193 journal articles, including thermal conductivity, electrical conductivity, Seebeck coefficient, and magnetic susceptibility. (See the Data Record section for the full list of recorded properties.) We facilitated the data extraction process using our web application Starrydata2. This application enables the extraction of data points from figures imaging temperature-dependent properties by simply clicking on the screen. This also allows for efficient conversion of the extracted image coordinates to their measurement values in the physical property space. The extracted data were managed with the compositional, sample preparation, and publication information in an easily accessible format. Once the extracted property values were registered, they could be visualized in the Starrydata2 system. The interactive visualization also aids human decision-making to improve the accuracy of data collection.

Starrydata2 implements a work environment that interoperates with WebPlotDigitizer, which was applied to the process of extracting numerical values from given images. First, a screenshot of the figure image from the source was captured and pasted into a designated area of WebPlotDigitizer. Subsequently, the reference points for each axis were specified by clicking on any two points within the x- and y-axes, and assigning the corresponding property values. Next, by clicking on each point from the property curves on the target graph, coordinate values were extracted and calculated with respect to the reference points for the x- and y-axes. This process was repeated for all samples in the graph, and the property coordinate data for each sample were stored and managed as a curve alongside sample information within Starrydata2.

The web application allows for the arbitrary specification of units (e.g., μΩcm for electrical resistivity is automatically converted to Ωm). Moreover, it facilitates unit conversion through internal unit and multiplier conversion functions. This ensures that recorded data, which may have different units or scales, can be managed consistently, thereby facilitating comparative studies and simplifying data utilization on the same scale.

Starrydata2 can also store sample information. Apart from chemical formulas, each sample is associated with the classification of QC and AC and experimental conditions.

## Data Records

### Composition dataset

The composition dataset provides a list of 915, 525 and 8 instances of QC, AC, and QC or AC, respectively. These were collected from 130 journal articles, consisting of 656, 463 and 8 unique compositions for QC, AC, and QC or AC, respectively. Each composition was linked to the literature information. The dataset is available in a CSV file named “composition_dataset.csv” on Figshare^30^. Table 1 lists the items present in the data table.

**Table 1:** List of items in the composition dataset.

Item	Data type	Description
ID	string	ID
Combination of elements	string	Combination of elements
Original formula	string	Compositional formula described in the literature
Alphabetical formula	string	Compositional formula of elements in alphabetical order
Number of elements	integer	Number of elements
Combination of elements	string	List of elements
Composition type	string	Composition type (e.g., nominal, alloy, analyzed)
QC, AC type	string	Structure type of QC or AC (IQC, IAC, DQC, or DAC)
Stability	string	Stability
Cluster type	string	Cluster type of QC or AC (Mackay, Bergman or Tsai)
Lattice type	string	Structure type of the lattice
Degree of approximant	string	Approximation degree of AC
Crystal system	string	Class of crystal structure
Analysis device	string	Devices used for compositional analysis
Phase information	string	Phase information
Sample shape	string	Shape of the sample
Space group	string	Space group
Pearson symbol	string	Pearson symbol
Lattice parameter	string	Lattice parameter
Heat treatment	string	Types of heat treatment in the sample fabrication
Heat treatment condition	string	Heat treatment conditions
Additional fabrication information	string	Additional information on the sample fabrication process
Solidification	string	Solidification processes and methods
Method	string	Method of sample fabrication
Note	string	Additional information
Data source	string	DOI or bibliographic information
Source type	string	Main: main subject of research in the article Reference: cited from a different article

### Phase region dataset

We used Scopus to narrow down the list of eligible articles to 218 papers authored by Benjamin Grushko obtained through the search query “TITLE-ABS-KEY (grushko)” as of May 26^th^ 2021. Subsequently, 327 Al-based ternary phase diagrams were extracted and saved as image files. Considering the temperature ranges of the sample preparation process and the range of phase diagrams (which may not cover the entire composition values of the three elements), 49 phase diagrams with unique combinations of the three elements were selected. If there were multiple phase diagrams for the same ternary system, preference was given to the one with a larger coverage area of the diagram or the one encompassing both QCs and Acs. From these phase diagram images, a total of 556 phase regions, comprising 21 QCs and 100 ACs, were extracted for a total of 16,208 reproduced compositions, including ordinary crystals and liquid phases. Table 2 presents the associated details. The phase region dataset named “phase_region_dataset.csv” is available on Figshare^30^.

**Table 2:** Details of the items in the phase region dataset.

Item	Data type	Description
ID	string	ID of the composition
Elements	list	Set of elements in the original phase diagram
Composition	key value	Composition ratio of the data point
Phase_type	string	Type of phases. IQC: icosahedral quasicrystal, IAC: icosahedral approximant, DQC: decagonal quasicrystal, DAC: decagonal approximant, CRY: crystal, LQD: liquid
Phase_symbol	string	Symbols denoted in the original phase diagram
Element1	string	The first element in the ternary system
Elm1_lower_range (atomic percent)	integer	Lower bound of the composition axis of Element1
Elm1_upper_range (atomic percent)	integer	Upper bound of the composition axis of Element1
Element2	string	The second element in the ternary system
Elm2_lower_range (atomic percent)	integer	Lower bound of the composition axis of Element2
Elm2_upper_range (atomic percent)	integer	Upper bound of the composition axis of Element2
Element3	string	The third element in the ternary system
Elm3_lower_range (atomic percent)	integer	Lower bound of the composition axis of Element3
Elm3_upper_range (atomic percent)	integer	Upper bound of the composition axis of Element3
DOI	string	DOI of the literature
Fig No.	string	Figure No. in the original article

### Properties dataset

In the properties dataset, data were extracted from 490 figures in 193 papers, including temperature-dependent observations of thermal conductivity, electrical properties, and magnetic properties, along with additional information on samples and bibliography. Table 3 provides the details for each item. Currently, a total of 1,449 temperature-dependent curves and 52,311 data points have been recorded. Table 4 presents the breakdown of the recorded curves. Data for 96 curves are included in the private version of Starrydata. The SID, sample_id, and figure_id of the corresponding data are appended with “hmt_”. The properties dataset is available in a CSV file named “properties_dataset.csv” on Figshare^30^.

**Table 3:** List of items in the properties dataset.

Item	Data type	Description
Composition	string	Composition of the sample
QC_or_AC_type	string	Type of QC or AC (IQC, IAC, DQC, DAC)
Degree_of_approximant	string	Approximation degree of AC
prop_x	string	Name of x axis
prop_y	string	Name of y axis
unit_x	string	Unit of x axis
unit_y	string	Unit of y axis
X	float	Value of x
Y	float	Value of y
SID	string	Unit of y axis
Sample_id	string	Sample ID in Starrydata2
Figure_id	string	Figure ID in Starrydata2
DOI	string	DOI of the literature

**Table 4:** Details of each property in the properties dataset.

Property	Number of curves
Electrical resistivity	736
Seebeck coefficient	227
Thermal conductivity	202
Magnetic susceptibility	73
ZT	72
Hall coefficient	40
Specific heat	32
Power factor	30
Specific heat capacity	19
Thermal diffusivity	18

## Technical Validation

### Composition dataset

To reduce recording errors and ambiguous wording in papers, issues were individually reviewed under the scrutiny of multiple experts when needed. To further ensure data accuracy, randomly sampled instances of the collected data underwent double-checking by the experts. If an error was found in a sampled record, the extracted information was reviewed and corrected as needed. A total of 330 compositions were revalidated during this exercise.

### Phase region dataset

**Fig. 3:**
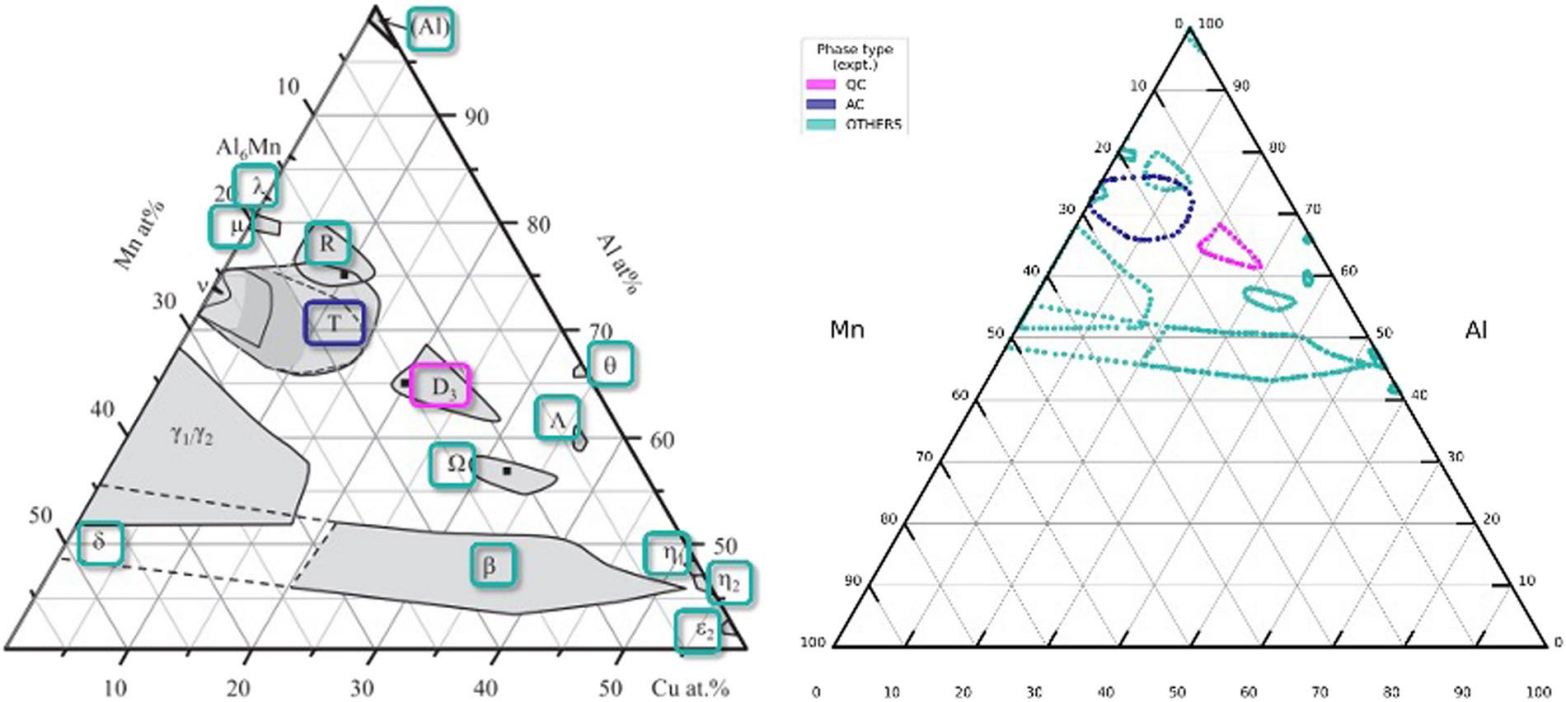
(**a**) Al-Mn-Cu ternary diagram sample^32^ (**b**). Image generated through processing the boundary point set of the phase region in the dataset. The black dots denote the extracted coordinate set, which is processed using a Python script for the visualization. The green, pink, and yellow regions indicate QC, AC, and other phases (ordinary periodic crystal or liquid phase), respectively.

The data in the phase region dataset were visually inspected on the screen. The data were provided as a set of discrete coordinates on a bounding region. The enclosed area defined by the coordinate set could be filled and visualized via a Python script. All generated images were verified to maintain consistency with their original figures, as depicted in Fig. 3.

**Fig. 4:**
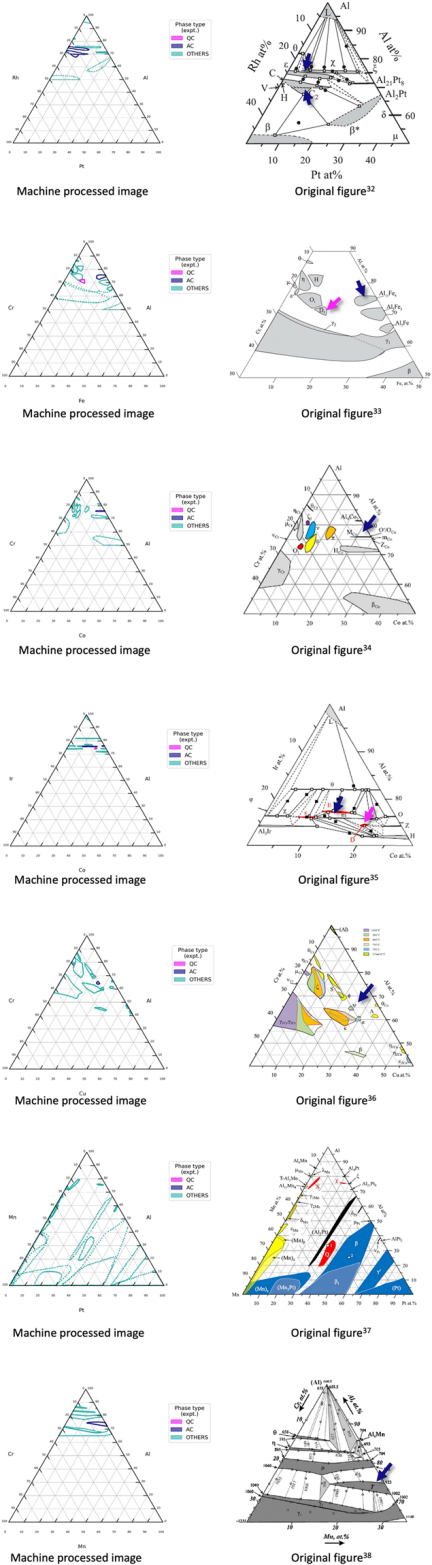
The left figure displays the extracted phase regions, where pink, navy, and light blue denote QC, AC, and crystalline phases, respectively. The corresponding original image is presented on the right side^33^–^39^.

As a part of validation, focusing on seven specific cases, the comparison between the extracted phase contour point sets and their original figures are shown in Fig. 4.

### Properties dataset

**Fig. 5:**
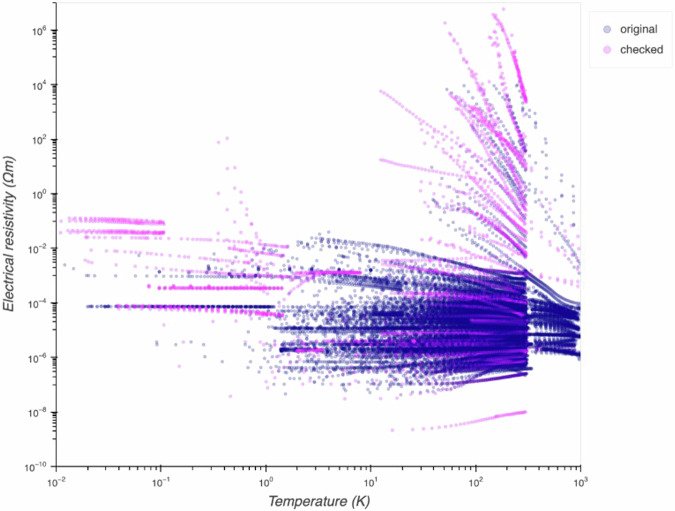
Temperature-dependent behavior of electrical resistivity is exhaustively visualized. Pink dots represent data points that underwent re-examination due to concerns regarding potential anomalies. 42 graphs containing curves that were visually identified as misaligned in behavior were subjected to re-examinations.

Property data were visualized using the Bokeh^31^ library in Python to double-check temperature-dependent behavior through expert discussions (Fig. 5). Temperature units such as 1/T (K^−1^) and 1000/T (K^−1^), physical property units, and physical property values that are not automatically converted were interactively corrected using Python scripts. Furthermore, the capitalization and orthographical variants were standardized for consistency.

Data extraction from images followed a standardized protocol in Starrydata2. We conducted a comprehensive test to measure the reading error of semi-automatic data extraction for several data collectors using WebPlotDigitizer (version 4.018). The results showed that the detection accuracy of the temperature-dependent property values for the test images was confined to approximately 0.30% of the overall width of the graph area. Since the data collectors in this study are participants of the Starrydata project and follow the same protocol, the data extraction accuracy is assumed to be comparable.

## Data Availability

The datasets of compositions, phase diagrams, and physical properties are available in Figshare^30^.
